# A New SIR-Based Sigmoid Power Control Game in Cognitive Radio Networks

**DOI:** 10.1371/journal.pone.0109077

**Published:** 2014-10-06

**Authors:** Yousef Ali Al-Gumaei, Kamarul Ariffin Noordin, Ahmed Wasif Reza, Kaharudin Dimyati

**Affiliations:** 1 Department of Electrical Engineering, Faculty of Engineering, University of Malaya, Kuala Lumpur, Malaysia; 2 Department of Electrical and Electronics Engineering, Faculty of Engineering, National Defence University of Malaysia, Kuala Lumpur, Malaysia; Southwest University, China

## Abstract

Interference resulting from Cognitive Radios (CRs) is the most important aspect of cognitive radio networks that leads to degradation in Quality of Service (QoS) in both primary and CR systems. Power control is one of the efficient techniques that can be used to reduce interference and satisfy the Signal-to-Interference Ratio (SIR) constraint among CRs. This paper proposes a new distributed power control algorithm based on game theory approach in cognitive radio networks. The proposal focuses on the channel status of cognitive radio users to improve system performance. A new cost function for SIR-based power control via a sigmoid weighting factor is introduced. The existence of Nash Equilibrium and convergence of the algorithm are also proved. The advantage of the proposed algorithm is the possibility to utilize and implement it in a distributed manner. Simulation results show considerable savings on Nash Equilibrium power compared to relevant algorithms while reduction in achieved SIR is insignificant.

## Introduction

The recent development in wireless networks applications and services led to a decrease in radio spectrum resources availability of the network. However, spectrum professional researchers and developers still found that licensed spectrum is underutilized in some locations and times [1]. Cognitive Radio (CR) is a promising technology that leads to optimal use of radio spectrum by allowing unlicensed users access to the unused parts (holes) of the licensed spectrum. Cognitive radios (CRs) that access the licensed spectrum are interference sources to other high priority licensed users. Therefore, CRs may not cause any undue interference to licensed users by keeping interference below the interference threshold level, commonly referred to as the “interference temperature” limit [2]. To reduce interference and guarantee achieving intended Quality of Service (QoS), an efficient power control algorithm is required to run in the CR devices.

Power Control (PC) in wireless networks has been widely considered in the past and recent years as an essential mechanism to maintain Signal-to-Interference Ratio (SIR). This in turn achieves the required Quality-of-Service (QoS) metrics, such as data rate and throughput [3]. In addition, PC can reduce the co-channel interference and extends the battery life in the Code Division Multiple Access (CDMA) systems, because each mobile device consumes minimum power needed to maintain the required SIR.

In the past, control theory has been considered in designing the power control algorithms in cellular networks and the main goal was to ensure that each mobile received Signal-to-Interference ratio (SIR) above a certain threshold value [4], [5], [6]. The most important and practical scheme of PC is distributed power control, in which each device can adjust power levels using only local measurements. The framework of uplink power control has been presented in cellular radio systems in [7], in which all users converge to the Pareto-optimal solution whenever they can achieve the required QoS that refers to SIRs. The most frequently referenced algorithm is the Constrained Distributed Power Control (CDPC), proposed by Grandhi and his co-authors [8], in which the upper limit of a transmission power was considered. The second-order constrained power control algorithm has been described in [9], in which the update of transmission power was depending on the past and current values of power. Authors in [10] designed a power control algorithm based on Linear Quadratic (LQ) control in order to achieve a faster convergence time and higher channel capacity. Similarly, authors in [11] proposed the exponential function of SIR in the power update equation to speed up the convergence.

The QoS objective of a terminal in wireless voice systems is to achieve a minimum acceptable SIR to maximize the number of conversations where the transmission errors are tolerable [12]. The QoS that refers to the SIR is no longer appropriate in wireless data networks because error-free communication had high priority [13]. Therefore, concepts of microeconomics and game theory have been used recently to define the users QoS in terms of utility (cost) function rather than SIR [14]. The utility function proposed in [13] was defined as a ratio between user throughput and its transmitted power and linear pricing terms have been included to improve the power control algorithm.

Various utility functions have been proposed in wireless data networks [15]–[17] as well as in cognitive radio networks [18]–[20]. Each user aims to maximize its own utility function value whenever possible in a distributed manner. The robustness of CDMA uplink power control game algorithm with respect to disturbance and time delays have been studied in [21]. In [22], authors proposed a new adaptive online power control scheme based on evolutionary game theory. The proposed scheme adaptively response to the constantly changing network environment and therefore, each user decides the transmit power level to control the co-channel interference efficiently. The reinforcement learning power control game is designed with low implementation complexity for CR networks in [23], in which interference channel and power strategy information were not required among CRs. Distributed resource allocation in primary and cognitive wireless networks has been studied in [24], in which CRs adjust their data rate and transmit power to maximize their own utility while the primary user has a fixed data rate and applies power control to achieve its target SIR. In [25], a distributed uplink resource allocation is proposed in multi-carrier wireless network, in which a user can optimize its own transmission rate by controlling it's transmit power and selecting a suitable access point. Energy efficient power control game in CDMA wireless networks over a frequency-selective channel with strict incomplete information has been considered in [26]. Authors in [27] developed an axiomatic framework for power allocation in cognitive radio networks that can achieve QoS protection of the licensed users, opportunism to the secondary users, admissibility to secondary users, and autonomous operation by the individual users. They found from the theoretical analysis and simulations that the Autonomous Interference-aware Power control (AIPC) can achieve such goals. The distributed power allocation for the secondary users in a cognitive radio using game model has been also considered in [28], in which the objective was to maximize each secondary user's throughput within its power budget and an interference constraint on the PU in its vicinity.

**Figure 1:**
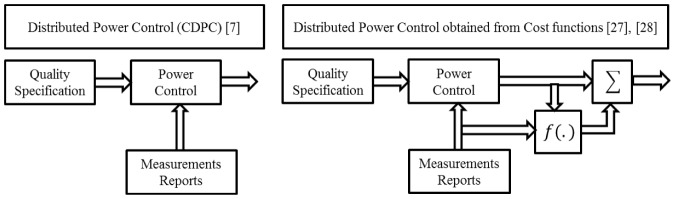
Power control blocks from a control perspective.

In the context of cost functions, Alpcan et al. [29] proposed a new cost function to design the SIR-based power control algorithm based on game theory framework. Alpcans cost function was logarithmically dependent on the SIR and linear in power and the objectives were to minimize the users cost. The cost function proposed in [30] consists of a weighted sum of linear power and square of SIR error. In [31], authors proposed a new cost function that consists of a weighted sum of linear power and hyperbolic of SIR error. Authors in [32] considered the power target in the cost function as well as the target SIR, in which each CR decreases its transmit power to be less than target power when the interference at the licensed user exceeds the interference temperature limit. In [33], authors proposed a doubled threshold adaptive algorithm based on cost function to optimize power allocation for users in CDMA cognitive radio networks. Power control algorithm formulas obtained by using cost functions [30], [31] consist of two terms, in which the first term is the same Distributed Power Control (DPC) formula and the second term depends on the function applied to SIR error, as shown in Figure 1.

In this paper, we proposed a novel cost function that consists of a weighted sum of power and square function of SIR error based on sigmoid function. As a rule, the cost function for the power control formula in this work also has two terms. The first term is similar to DPC algorithm and the second term is the price term. Important features of the proposed sigmoid power control scheme are: (i) the ability to maintain the required QoS of all CRs efficiently with insignificant reduction in SIR, (ii) the algorithm can be practically implemented in a distributive manner without requiring additional information from BS, and (iii) a significant decrease and better fairness in mobile power consumption. The novelty of the proposed sigmoid power control scheme is the sigmoid-based cost function. The choice of the proposed sigmoid-based cost function is the key to enable each CR to choose its transmitting power efficiently. It guides cognitive users to achieve lower power consumption level compared to other algorithms. Furthermore, we explained the variation between power control algorithms obtained from control theory and game theory, and presented these algorithms depending on the channel status. On the other hand, the proposed algorithm can be practically implemented in a distributive manner without requiring additional information.

The rest of this paper is as follows: Section 2 describes the system model of cognitive radio, the distributed power control and the game model of the proposed power control algorithm. Section 3 presents the numerical results and discussion. Limitation of this study and future works are discussed in Section 4 and the conclusion is presented in Section 5.

## Materials and Methods

### System model

**Figure 2:**
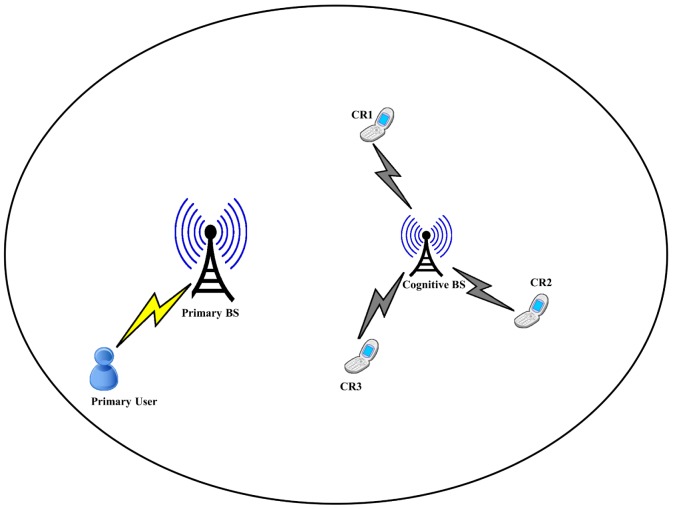
System model of cognitive radio networks.

As shown in Figure 2, we consider a single cell wireless cognitive radio CDMA network in which 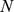 CRs share the same licensed spectrum with primary users, and one CR base station. Let 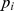 be the transmit power of *i*th cognitive user and 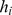 is the attenuation from *i*th cognitive user to the base station. The attenuation is computed from the distance 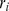 between the *i*th CR and the cognitive base station to be 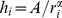 with neglected shadowing and fast fading effects, where *A* is a constant gain and *α* is the path loss factor that is usually between 2 and 6. In this paper, it is assumed that CRs are stationary; and only the uplink power control case is considered. The SIR of *i*th CR can be defined as 
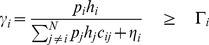
(1)


where 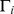 is the target SIR, 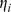 is the background noise, and 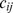 is the correlation coefficient. The sum of interference including noise in the denominator of Equation (1) can be denoted as 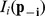, hence Equation (1) can be rewritten as: 
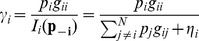
(2)


The subscript 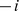 indicates the interference that depends on the power of all users except the *i*th user. Comparing between Equations (1) and (2) produce the following equation: 
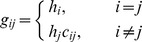
(3)


In spectrum trading, the objective of primary system that owns the spectrum rights is to maximize its revenue (profit) by sharing unused parts of spectrum with large number of CRs. The profit maximizing objective should be set under the constraint on limited performance degradation of primary users or the interference temperature limit [3]. The interference temperature constraint is expressed as 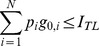
(4)


where 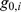 is the channel gain from the transmitter of cognitive radio *i* to the measurement point of the primary system, and 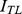 is the interference temperature limit. Due to the competition of many cognitive radio networks to share the unused spectrum, it is assumed that the primary system has an intelligent admission control policy that will accept only the lowest interference CR network. Therefore, a new power control algorithm for cognitive radio networks has been considered in this work to guarantee lower interference and thus obtaining high priority in the admission control of the primary system.

To facilitate the presentation and analysis, we also further introduce a user-specific notation 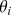 as a ratio of interference to the path gain of cognitive user *i* (as in [16]), shown in the following equation: 
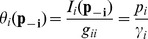
(5)


The value of 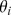 represents the channel status of cognitive user *i*; a higher interference and a lower path gain results in a higher 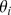. The poor channel of cognitive user *i* results in a higher value of 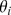, while the good channel resulted in a lower value of 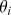. In [7], the user *i* maintains its SIR at a target level 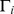, and the power update formula depends on the previous values of power and SIR as 
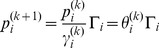
(6)


**Figure 3:**
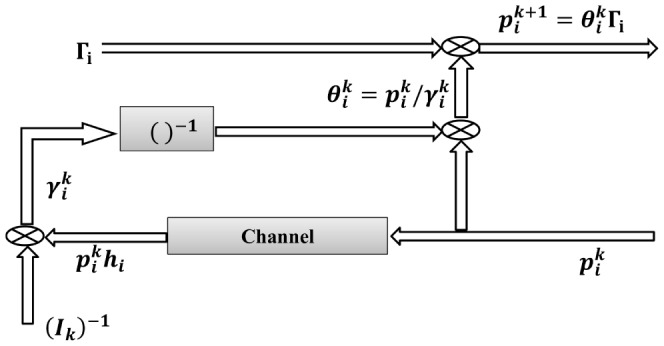
Control Block diagram of the CDPC.

The unconstrained power control in Equation (6) converges to a fixed point if the target SIR vector is feasible and all users can achieve their target SIR with minimal transmit power. On the other hand, if the target SIR vector is infeasible, then there is no transmit power vector that can satisfy SIR requirement for all users. Distributed power control has been improved in [8] to the constrained power control in order to solve the problem of divergence in infeasible case, that is, 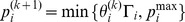
(7)


where 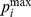 is the maximum constrained transmit power. The control block diagram of the CDPC is shown in Figure 3, as in [5].

### Power Control Based on Game Model

The interaction behavior among CRs requires a suitable framework for analysis. Recently, game theory has been considered as the most efficient tool for analyzing the interaction of decision makers. Game model usually consists of three basic elements: (i) players or decision makers of the game, (ii) strategy or action space, and (iii) utility or cost function. Each player in game selects its action from action space to maximize (or minimize cost) its own utility in a selfish manner. In CR networks, CR users can be considered as the decision makers of the game, network resources (power, data rate, etc.) are the strategy spaces of the game, and the utility function represents the preference (required QoS) of CR users. In this paper, we defined the non-cooperative power control game of a CR system as follows: 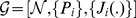
(8)


where 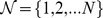 is the index set of players (CRs), 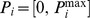 represents the transmission power strategy set of user *i*, and 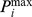 is the maximum transmission power of user *i*. The cost function of user *i* is referred to as 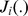, in which each CR tries to minimize its own cost function in a distributive manner.

### Cost Function and Nash Equilibrium Derivation

Several cost functions have been proposed to solve the problem of power control in wireless data networks. Given a reference target SIR 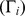, power control is used to maintain this SIR based on the feedback of the error 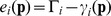. We assume that the cost function of *i*th cognitive user is 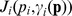, where the power vector is 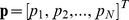. The Nash equilibrium point(s) means that no user can improve its individual cost function unilaterally. Mathematically, for all 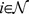


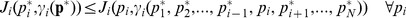
(9)


Thus, the proposed cost function can be written as 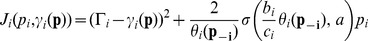
(10)


where 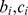 are non-negative weighting factors, and *a* is the sigmoid factor. The function 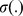 represents the sigmoid function, which is defined as 
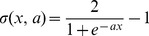
(11)


The power update formula is obtained by differentiating the cost function with respect to power and equating it with zero, 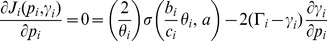


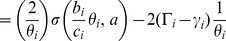
(12)


Rearranging terms of Equation (12) yields 
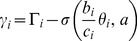
(13)


Substituting for 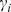 in Equation (2), we can express the power update formula as 
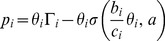
(14)


### Power Control Algorithm

**Figure 4:**
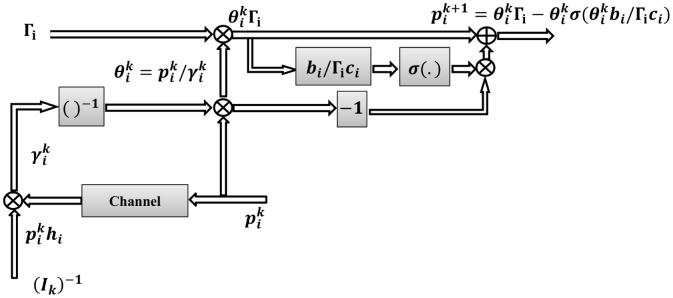
Control Block diagram of the proposed sigmoid power control.

According to Equation (14), each CR user can update its power level using only the knowledge of its own interference level; therefore, this method can be implemented in a distributed manner. We assume that the algorithm is updated every step and depending on the measured interference, the proposed power update formula can be written as 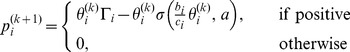
(15)


where 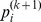 is the power level of the *i*th user at the 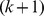
th step and 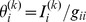 is the channel status of the *i*th user that depends on the measured interference at the *k*th step of the algorithm.The control block diagram of the sigmoid power control is shown in Figure 4.

### Convergence

In [34], the author shows that if the algorithm 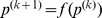 converges to a fixed point, the function *f* should satisfy the following three conditions:

1. Positivity 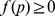,

2. Monotonicity 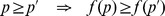,

3. Scalability 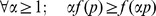.

First, we prove the positivity condition. Since 
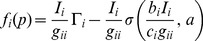
(16)


Therefore, if we want 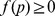, it needs 
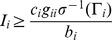
(17)


Since 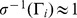, if we choose a proper value for 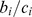, the positivity condition can be easily met. The monotonicity condition can be proved by increasing the best response function with respect to 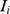. By differentiating equation (16) with respect to 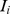, we get 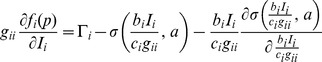
(18)


Using inequalities 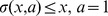 and 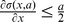, for monotonicity, we should have 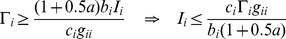
(19)


Finally, condition of the scalability in our method can be written as 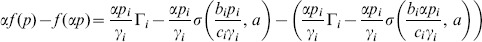


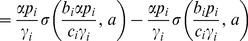
(20)


Since 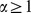, we have 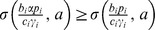. Therefore, for scalability, the positivity can be met and it is sufficient. From above analysis and according to the parameters, we can conclude that the power control function is a standard function and the algorithm converges to a unique Nash Equilibrium point.

### Existence of Nash Equilibrium

In this section, a solution is presented for the Nash algorithm algebraic equations to guarantee the existence of a unique solution to the power update equation. The result is decided by using the Implicit Function Theorem [35]. From Equation (14) and by using 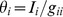, we obtained the following algebraic equations: 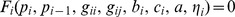


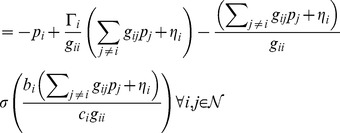
(21)


According to the Implicit Function Theorem, the Jacobian matrix 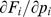 must be a non-singular at the point of the existence. Since, 
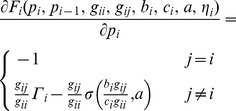
(22)


The corresponding Jacobian matrix has −1 on the main diagonal and other elements are determined by 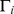. The value of the Jacobian matrix is relevant to 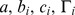, and 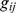. The value of the sigmoid function is relevant to the parameters 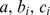, which is not too large and the value of 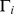 is also not too large. The path loss 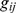 is very small in practice, which depends on the distance of CR to the base station. Thus, the Jacobian matrix is a non-singular and this proves the existence of Nash Equilibrium.

### Numerical Results and Discussion

**Figure 5:**
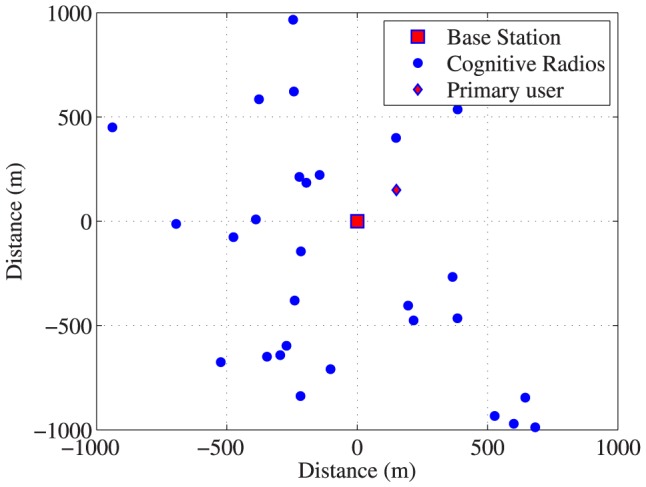
Random distribution of 30 cognitive users and one primary user.

In this section, we compare the performance of our proposed algorithm with CDPC algorithm [8], Norm-2 algorithm [30], and Hyperbolic algorithm [31], [36]. The cost functions and power control algorithm formulas that have been used in the comparison are explained in Table 1. Initially, we demonstrate the system environment to which our algorithm and above existing algorithms are applied. We consider a 2000 m ×2000 m cell with one cognitive base station located at the center and 30 CR users located randomly given by a uniform distribution. A simple sketch of the system model is shown in Figure 5, in which the primary user may be interfered with CR users. In this paper, the fast fading, shadowing, and interference from the adjacent cells were neglected. The background noise power is considered to be 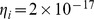 W. The channel gain was computed according to 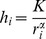
(23)


**Table 1:** Cost functions and power control formulas used in the simulation comparison.

Algorithm	Cost function	PC formula
CDPC [8]	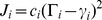	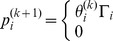
Norm-2 Algorithm [30]	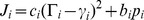	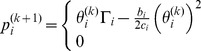
Hyperbolic Algorithm [31], [36]	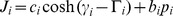	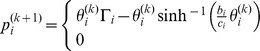

where 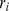 is the distance between the *i*user and base station, *α* is the path loss exponent, which is supposed to be 4, and 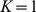 is a constant. The processing gain is set to 128.

### Effect of Channel Status 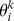 to the Next Step of Power

**Figure 6:**
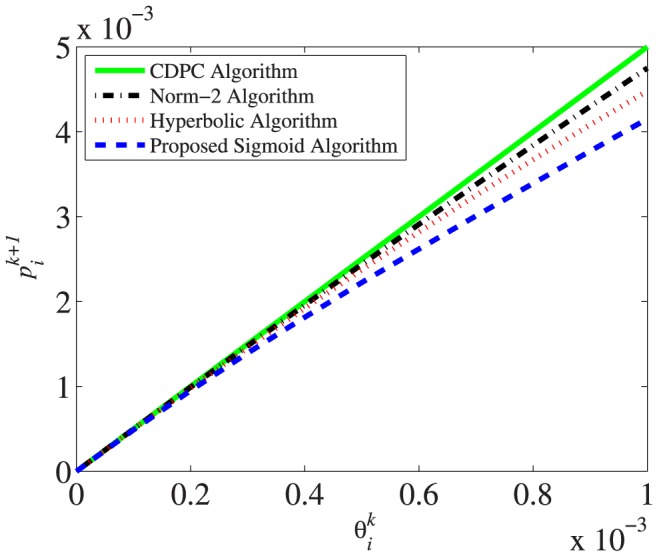
Comparison of power update for a range of channel status.

We set the value of target SIR as 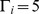, the ratio of weighting factors 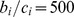, and arranged the channel status value from 0 to 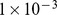. Figure 6 shows the values of power 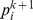 according to 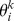, and it is found that our proposed sigmoid power control has the lowest power compared to other algorithms.

### Fully-loaded Power and SIR

**Figure 7:**
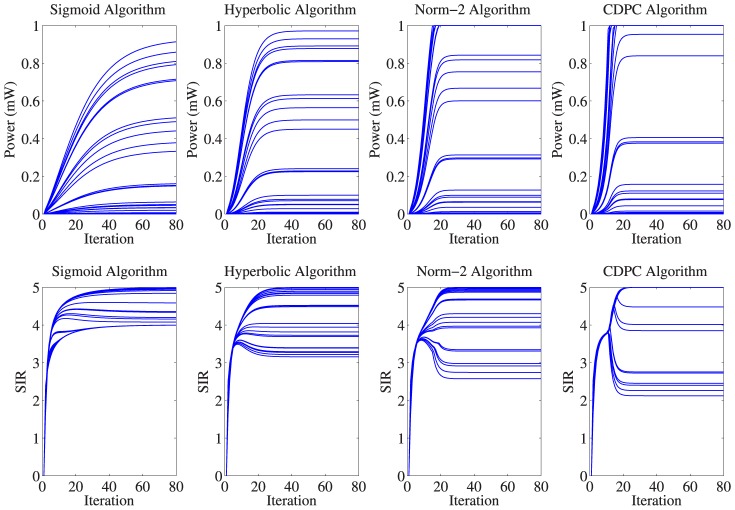
Performance comparison of proposed sigmoid algorithm and other algorithms for 30 CRs.

In this simulation, all cognitive users start with initial power 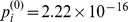 W, and we used the target value of SIR as 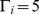. The values of the non-negative weighting factors are 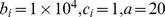, and the maximum constraint power of all users 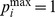 mW. We evaluated all algorithms using MATLAB. The results of power and SIR for the four schemes of all CRs are shown in Figure 7. It is observed that the proposed sigmoid algorithm guarantees that all users can achieve their target SIR with the reduction of no more than 20% 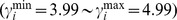, while for the other algorithms, some users may obtain SIR reduction of more than 50%. On the other hand, the maximum power used by the farthest user in our proposed sigmoid algorithm is 0.915 mW, while in hyperbolic algorithm is 0.972 mW, and the others reach the maximum power of 1 mW. The number of CRs that reach 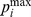 is demonstrated in Table 2.

**Table 2:** Numerical results obtained from the simulation.

Algorithm	min SIR	max SIR	No of CRs reach 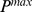	No of CRs with 25% reduction in SIR
CDPC [8]	2.123	5	9	24
Norm-2 [30]	2.575	5	6	24
Hyperbolic [31], [36]	3.157	5	0	22
Proposed Sigmoid	3.996	4.995	0	30

**Figure 8:**
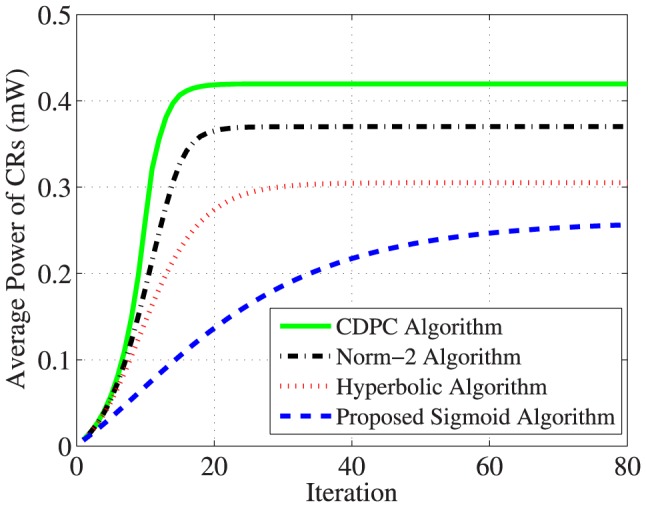
Comparison of average power for 30 CRs.

As shown in Figure 8, the average power computed by the proposed sigmoid algorithm is significantly saved, i.e., 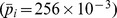 mW, while the other algorithms are 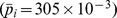 mW in Hyperbolic algorithm, 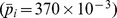 mW in Norm-2 algorithm, and 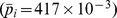 mW in CDPC algorithm. The reason for improvement in power saving refers to the term 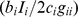 that was introduced in Norm-2 algorithm and replaced by 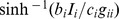 in the hyperbolic algorithm and replaced by 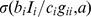 in our proposed sigmoid algorithm. If we compare these terms, it will be observed that 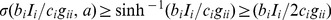
(24)


**Figure 9:**
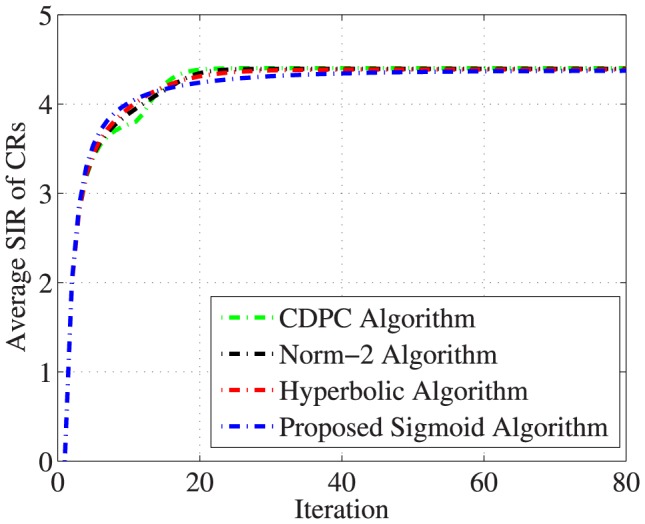
Comparison of average SIR of 30 CRs.

On the other hand, Figure 9 shows the insignificant difference in the reduction of the average SIR, in which the average SIR of the proposed sigmoid algorithm is 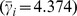, while other algorithms are found to be 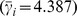 in Hyperbolic algorithm, 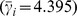 in Norm-2 Algorithm, and 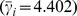 in CDPC algorithm.

### Impact of Noise

**Figure 10:**
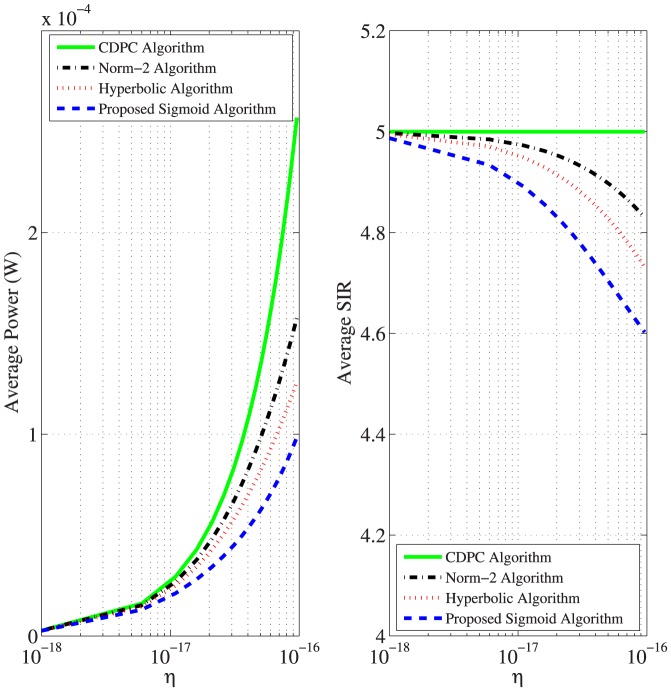
Performance comparison of average power and SIR for 25 CRs for a range of noise values.

We tested the algorithm with the condition of 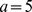, and 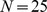 CRs, with the same values of parameters that were used in previous test. The range of noise power is from 10^−18^ W to 10^−16^ W. As shown in Figure 10, the average power increases and the SIRs decrease with the increase of noise for all algorithms because power is proportional to noise while SIR is inversely proportional to noise as in Equation (1). The proposed sigmoid algorithm provides significant savings of power in high noise environments, while the reduction in SIR is insignificant compared to other algorithms.

### Limitations and Future Works

The main objective of this work was to maintain the required QoS of CRs with significant decrease and better fairness in mobile power consumption in order to mitigate the aggregate interference. The iteration method of the proposed sigmoid power control algorithm was the Jacobi iteration which is a fixed-point iterative method. The fixed-point iterative method has slow convergence speed, and the rate of convergent is linear. It is anticipated that the convergence speed of the sigmoid power control algorithm in Equation (15) can be improved and accelerated using a more advanced iterative methods, like newton or secant methods that have quadratic and super-linear rate of convergent, respectively. The idea of newton method is to replace nonlinear function 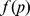 in Equation (16) with its linear approximation using Taylor series as [35] [37]

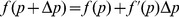
(25)


In order to find the root of this function, we set the linear approximation to 0: 
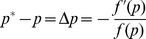
(26)


Therefore, for each iteration, the power iterative formula rule becomes: 
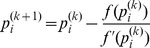
(27)


On the other hand, cognitive radios were distributed randomly in the area of the cell and each CR has a different value of path gain that depends on its distance from the base station. The sigmoid power control algorithm has been applied to all users with different path gains in a static scenario to show the effectiveness of the algorithm. Static scenario was considered in our work in order to match with the other algorithms that used the same scenario. Since the track of CRs is important in real systems, we will consider the proposed sigmoid algorithm in a dynamic scenario for our future work where the predictability of interference power and path gain would be the main perspectives.

## Conclusion

Having studied several power control algorithms based on different cost functions, a new cost function for SIR-based power control algorithm in cognitive radio networks has been proposed. The proposed power control algorithm achieves a significant reduction in power at approximately the same level of average SIR. This will enable the proposed algorithm to serve more CRs and thus achieve the optimal exploitation of the spectrum with the least amount of interference. In addition, the proposed algorithm results in better fairness, in which all users meet their SIR constraints without transmitting at high power levels. The proposed method can be applied to the uplink of two tier femtocell networks using the Stackelberg game approach. However, an efficient pricing technique would be required to manage the cross-tier interference.
